# Automated sign language detection and classification using reptile search algorithm with hybrid deep learning

**DOI:** 10.1016/j.heliyon.2023.e23252

**Published:** 2023-12-08

**Authors:** Hadeel Alsolai, Leen Alsolai, Fahd N. Al-Wesabi, Mahmoud Othman, Mohammed Rizwanullah, Amgad Atta Abdelmageed

**Affiliations:** a Department of Information Systems, College of Computer and Information Sciences, Princess Nourah bint Abdulrahman University, P.O. Box 84428, Riyadh 11671, Saudi Arabia; b Department of Computer Science, College of Science & Art at Mahayil, King Khalid University, Saudi Arabia; c Department of Computer Science, Faculty of Computers and Information Technology, Future University in Egypt New Cairo 11835, Egypt; d Department of Computer and Self Development, Preparatory Year Deanship, Prince Sattam bin Abdulaziz University, AlKharj, Saudi Arabia

**Keywords:** Sign language, Deep learning, Computer vision, Reptile search algorithm, Intelligent models

## Abstract

Sign language recognition (SLR) contains the capability to convert sign language gestures into spoken or written language. This technology is helpful for deaf persons or hard of hearing by providing them with a way to interact with people who do not know sign language. It is also be utilized for automatic captioning in live events and videos. There are distinct methods of SLR comprising deep learning (DL), computer vision (CV), and machine learning (ML). One general approach utilises cameras for capturing the signer's hand and body movements and processing the video data for recognizing the gestures. One of challenges with SLR comprises the variability in sign language through various cultures and individuals, the difficulty of certain signs, and require for realtime processing. This study introduces an Automated Sign Language Detection and Classification using Reptile Search Algorithm with Hybrid Deep Learning (SLDC-RSAHDL). The presented SLDC-RSAHDL technique detects and classifies different types of signs using DL and metaheuristic optimizers. In the SLDC-RSAHDL technique, MobileNet feature extractor is utilized to produce feature vectors, and its hyperparameters can be adjusted by manta ray foraging optimization (MRFO) technique. For sign language classification, the SLDC-RSAHDL technique applies HDL model, which incorporates the design of Convolutional Neural Network (CNN) and Long-Short Term Memory (LSTM). At last, the RSA was exploited for the optimal hyperparameter selection of the HDL model, which resulted in an improved detection rate. The experimental result analysis of the SLDC-RSAHDL technique on sign language dataset demonstrates the improved performance of the SLDC-RSAHDL system over other existing DL techniques.

## 1 Introduction

Sign language is a computer vision-based comprehensive complex language that captivates signs formed by the actions of hands in association with facial expressions [1]. It is a natural language employed by an individual with less or no hearing intelligence for communication. Sign language can be implemented for communicating words, letters, or sentences by employing diverse gestures of the hands [2]. This kind of communication makes it simple for hearing-challenged individual to express their opinions and assist in linking the communication gap amongst normal and hearing-challenged individuals. People have adapted to sign language for communicating since the antique period [3]. Hand signs are as old as human civilization itself. Hand gestures are specifically advantageous in expressing any emotion or word to communicate. Hence, humans around the globe employ gestures from hand regularly in expressing themselves spite the creation of writing conventions [4]. Recently, much study has been continuing in emerging systems that are able to classify gestures of diverse sign languages as provided class. Such systems have found applications in robot controls, natural language communications, virtual reality environments, and games [5]. The automated identification of human gestures is a convolutional multi-disciplinary issue that has not yet been totally resolved. In recent years, a count of methods can be employed that involve the implementation of ML procedures for sign language identification [6]. Since the beginning of Deep Learning (DL) methods, there have been attempts to identify human gestures.

To identify gestures, diverse aspects like articulated models and hand-crafted spatio-temporal descriptors were employed together with gesture classifiers, conditional random fields [7], hidden Markov models, and Support Vector Machines (SVM) have been extensively employed. But categorization of signs is unforeseeable under changing illumination conditions, and from diverse subjects is still a threatening issue [8]. An instinctive approach for producing interfaces is to look at the user's muscle activity. The device can record this action by employing a camera [9]. This recorded imagery can be recognized by DL algorithms to determine the gesture. In recent times, categorization with DCNN networks has been efficient in several identification challenges [10]. Multi-column DCNNs that use several similar networks have been demonstrated to enhance recognition rates of single networks.

This study introduces an Automated Sign Language Detection and Classification using Reptile Search Algorithm with Hybrid Deep Learning (SLDC-RSAHDL). In the SLDC-RSAHDL technique, MobileNet feature extractor is utilized to produce feature vectors, and its hyperparameters can be adjusted by manta ray foraging optimization (MRFO) system. For sign language classification, the SLDC-RSAHDL technique applies HDL model, which incorporates the design of Convolutional Neural Network (CNN) and Long-Short Term Memory (LSTM). At last, the RSA was exploited for the optimal hyperparameter selection of the HDL model, which resulted in improved detection rate. The experimental result examination of the SLDC-RSAHDL algorithm was executed on sign language database.

## 2 Literature review

Pandey et al. [11] proposed a novel Feed Forward Neural Network (FFNN) model system that can automatically identify sign language to help normal humans in more efficient communication with impaired visually, hearing-wise, or speech-wise. This scheme recognized the hand gesture aspect point extraction given with FF point extraction given with FFNN. Hand gesture recognition with voice process scheme by implementing Hidden Markov Model (HMM) is employed to deliver communication for normal and dump individuals. In Ref. [12], a new outline is suggested for gesture-autonomous sign language identification by employing several DL constructions containing hand semantic segmentation, Deep Recurrent Neural Network (DRNN), and hand shaped factor depiction. Abstracting hand shaped aspects is performed by implementing a single layer Convolutional Self-Organizing Map (CSOM) rather than depending on transfer learning (TL) of pre-trained CNNs (DCNNs). The series of abstracted aspect vectors is later identified by implementing BiLSTM-RNN.

In [13], a two-stream CNN (2 S–CNN) framework was suggested to identify the American Sign Language (ASL) hand signs founded on multi-modal (RGB and depth) data fusion. Initially, the hand sign information was improved to eliminate the impact of noise and background. Next, hand sign RGB and depth features are abstracted for hand sign detection by corresponding CNNs on 2 streams. Lee et al. [14] suggest an ASL learning application model. This application will be a whack-a-mole gaming with an embedded real time gesture identification scheme. As both dynamic and static gestures (J, Z) are present in ASL alphabetical system, LSTMRNN with KNN technique is accepted as the categorization technique is founded on management of a series of inputs. Features like angles amongst fingers, distance amongst finger positions, and sphere radius are abstracted as input for the categorization prototype.

Rastgoo et al. [15] suggest a new DL-founded pipeline construction for effective instinctive hand gesture language identification by implementing 2DCNN, Single Shot Detector (SSD), 3DCNN, and LSTM from RGB input videos. The authors employ a CNN-founded prototype that evaluates the 3D hand keypoint from 2D input segments. Das et al. [16] suggested a fusion porotype comprising deep TL founded on CNN with an RF categorizer for the instinctive identification of Bangla Sign Language (BSL) (numeric and alphabetical symbols). 'Ishara-Bochon' and 'Ishara-Lipi' are both datasets of secluded numeric and alphabetical symbols, corresponding to the initial comprehensive multi-purpose open-access dataset for BSL. Also, the authors suggested a background elimination protocol that eliminates needless aspects from the gesture imageries. The authors [17] suggest a Fully Convolutional Network (FCN) for online SLR to simultaneously learn temporal and spatial aspects from feebly interpreted video series with sole sentence-level explanations provided. A Gloss Feature Enhancement (GFE) segment is presented in the suggested networks to apply better series orientation learning.

## 3 The proposed model

**Fig. 1:**
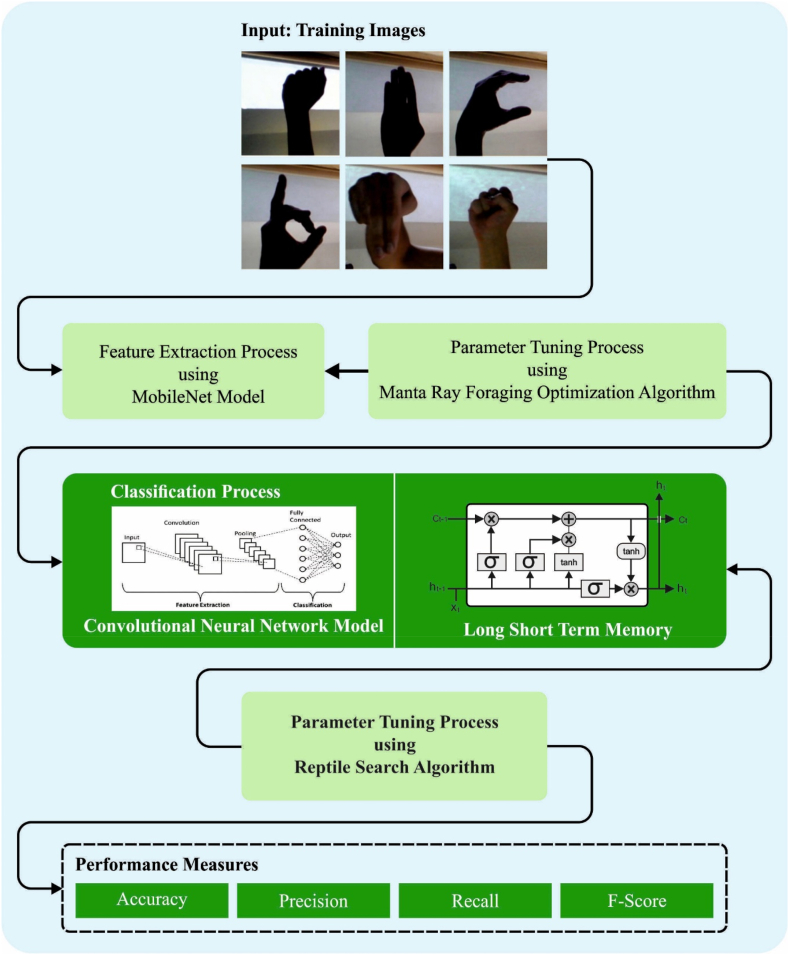
Overall flow of SLDC-RSAHDL approach.

In this article, we have introduced a new SLDC-RSAHDL technique for automated detection and classification of sign language using the DL and metaheuristic optimization algorithms. It follows a four stage process: MobileNet feature extraction, MRFO based hyperparameter tuning, HDL based sign language recognition (SLR), and RSA based parameter tuning. Fig. 1 signifies the overall flow of SLDC-RSAHDL approach.

### 3.1 Feature extraction using MobileNet

The basic principle of lightweight model is to develop effective network computation for convolution models that could minimalize the number of parameters and the computation time while guaranteeing the detection performance. Sifre, in the US in 2014, first proposed the MobileNet model, which was the depth‐separable convolution that splits the typical convolutional layer into point‐wise and depth‐wise convolutional layer separable convolutional layer that implies the summation and convolution in the classical convolutional model are divided as, such that the computation speed is improved increased and thus, the amount of weight parameters evaluated by the network could be decreased considerably [18].

Consider that the length and width of output and input are constant and that the number of channels M, input is a feature map of length $D_{F}$ and width $D_{F}$, later a convolutional kernel of height $D_{K}$ and width $D_{K}$, the typical convolution will output a number of channels N, feature map of length $D_{K}$ and width $D_{K}$. Set this to G; the typical convolution is $D_{F}xD_{F}xMxNxD_{K}D_{K}$. This convolution process was mathematical process written as:

(1) $$G_{k,l,n}=\sum_{i,j,m}K_{i,j,m,n∙}F_{k+i-1,l+j-1,m}\;$$

The computation of every $\hat{G}$ needs the sum of each m. Depth‐separable convolution to take out the m alone.

Later depth separable convolutional layer splits the classical convolution kernels into summation and convolution parts. In such cases, the pointwise convolution map has single parameter, the amount of resultant features N, whereas the depth convolutional map has three variables, the amount of input features M, the length $D_{K}$ and the width $D_{K}$. The original 4 parameters were split into 1 and 3 parameters; hence it can be mathematical model has been changed.

(2) $$\hat{G}=\sum_{m}{\sum_{i,j}\hat{K}}_{i,j,m}F_{k+i-1,l+j-1,m}\;$$

(3) $$G_{k,l,n}=\sum_{m}{\hat{G}}_{k,l,m}\cdot {\bar{K}}_{m,n}\;$$

Where K represents the convolutional kernel for pointwise convolutional and $\hat{K}$ represents the convolutional kernel for depthwise convolutional.

(4) $$G_{k,l,n}=\sum_{m}{\sum_{i,j}\hat{K}}_{i,j,m},F_{k+i-1,l+j-1,m}\cdot {\bar{K}}_{m,n}\;$$

For such reasons, it is easier to ensure for the depth separable convolutional, the amount of convolution execution is evaluated in 2 stages. Initially, $MD_{K}xD_{K}$ matrices moved $D_{F}xD_{F}$ times; next, N 1x 1xM convolutional kernels moved $D_{F}xD_{F}$ times; hence the overall amount of convolutional executions can be attained by adding the number of depth‐separable convolutional and the abovementioned two executions. The amount of computation is $D_{F}xD_{F}xMxD_{F}xD_{F}+1x1xMxNxD_{F}xD_{F}$, The ratio of computation work of the depth separable convolutional layer to the typical convolutional can be given as follows:

(5) $$\frac{D_{K}\times D_{K}\times M\times D_{F}\times D_{F}\times M\times N\times D_{F}\times D_{F}}{D_{K}\times D_{K}\times D_{F}\times D_{F}\times M\times N}=\frac{1}{N}+\frac{1}{D_{K}^{2}}\;$$

The abovementioned formula demonstrates that the computation reduction is positively related to $D_{K}$ and N. Furthermore, the convolution kernels of the depth convolutional layer in MobileNet are known to be 3x3, and during their implementation, the computation of depth separable convolutional layer is 1/8 to 1/9 of that of the typical convolutional, thereby accomplishing the drive of enhancing the computational rate of network structure.

### 3.2 Hyperparameter tuning using MRFO algorithm

For hyperparameter tuning process of the MobileNet algorithm, the MRFO technique was employed. The MRFO algorithm simulates three foraging performances for upgrading the solution position [19]. The foraging performances like cyclone, somersault, and chain. The mathematical process for every foraging performance is described below:

Chain foraging: The foraging chain has been developed if manta rays arrange head‐to‐tail. In each iteration, an optimum solution was utilized for updating every individual. The subsequent mathematical model can demonstrate it:

(6) $$x_{i}^{d}\left(t+1\right)=\left\{ \begin{array}{l} x_{i}^{d}\left(t\right)+r\cdot \left(x_{best}^{d}\left(t\right)-x_{i}^{d}\left(t\right)\right)+\alpha \cdot \left(x_{best}^{d}\left(t\right)-x_{i}^{d}\left(t\right)\right),i=1\\ x_{i}^{d}\left(t\right)+r\cdot \left(x_{i-1}^{d}\left(t\right)-x_{i}^{d}\left(t\right)\right)+\alpha \cdot \left(x_{best}^{d}\left(t\right)-x_{i}^{d}\left(t\right)\right),i=2\ldots ,N \end{array}\right.$$

$$\alpha =2\cdot r\cdot \sqrt{|\;\log\;\left(r\right)|}$$

Whereas N signifies the dimensional of populations, r denotes the random vector among 0 and 1, $x_{i}^{d}\left(t\right)$ refers to the $i^{th}$ individual's position at $t^{th}$ iteration, α implies the weighted coefficient, and $x_{best}^{d}\left(t\right)$ stands for the plankton with maximal concentration (an optimum solution gained so far).

Cyclone foraging: If the manta rays spot food, they can generate a lengthy foraging chain and therefore swim for receiving the food. The subsequent mathematical formula defined the cyclone foraging performance:

(7) $$x_{i}^{d}\left(t+1\right)=\left\{ \begin{array}{l} x_{best}^{d}\left(t\right)+r\cdot \left(x_{best}^{d}\left(t\right)-x_{i}^{d}\left(t\right)\right)+\beta \cdot \left(x_{best}^{d}\left(t\right)-x_{i}^{d}\left(t\right)\right),i=1\\ x_{best}^{d}\left(t\right)+r\cdot \left(x_{i-1}^{d}\left(t\right)-x_{i}^{d}\left(t\right)\right)+\beta \cdot \left(x_{best}^{d}\left(t\right)-x_{i}^{d}\left(t\right)\right),i=2\ldots ,N \end{array}\right.$$

$$\beta =2e^{r_{1}\left(T-t+1/T\right)}\cdot \sin\left(2\pi r_{1}\right)$$

In which β and T signify the weighted factor and maximal iteration count correspondingly, and $r_{1}$ denotes the random value among zero and one.

The exploration process is utilized for improving the algorithm by utilizing the subsequent mathematical process:

(8) $$x_{rand}^{d}=Lb^{d}+r\cdot \left(Ub^{d}-Lb^{d}\right)\;$$

(9) $$x_{i}^{d}\left(t+1\right)=\left\{ \begin{array}{l} x_{rand}^{d}\left(t\right)+r\cdot \left(x_{rand}^{d}\left(t\right)-x_{i}^{d}\left(t\right)\right)+\beta \cdot \left(x_{rand}^{d}\left(t\right)-x_{i}^{d}\left(t\right)\right),i=1\\ x_{rand}^{d}\left(t\right)+r\cdot \left(x_{i-1}^{d}\left(t\right)-x_{i}^{d}\left(t\right)\right)+\beta \cdot \left(x_{rand}^{d}\left(t\right)-x_{i}^{d}\left(t\right)\right),i=2\ldots ,N \end{array}\right.$$

whereas $x_{rand}^{d}$ denotes the random position from the searching space, and $Ub^{d}$ and $Lb^{d}$ imply the lower and upper limits of $d^{th}$ dimensional correspondingly

Somersault foraging: The food position at this point was considered as pivot, whereas all the individuals performed to swim near or around the pivot and afterwards somersaults to a novel position. The equivalent mathematical formula is offered as depicted:

(10) $$x_{i}^{d}\left(t+1\right)=x_{i}^{d}\left(t\right)+S\cdot \left(r_{2}\cdot x_{best}^{d}-r_{3}\cdot x_{i}^{d}\left(t\right)\right),i=1,\ldots ,N$$

Whereas the somersault factor was defined by S, and $r_{2}$ and $r_{3}$ signify the random numbers among zero and one.

### 3.3 Sign language classification using optimal HDL model

**Fig. 2:**
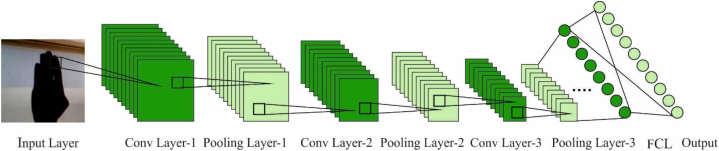
Structure of CNN.

In this work, the classification of signs takes place by the HDL model. For two major reasons, CNN provides better accuracy in pattern recognition and classification. Primary, its structure was highly relevant for determining local connections amongst data points; next, it decrease the amount of network parameters [20], thus resulting in a low computation difficulty than traditional plain neural network architecture. Fig. 2 displays the structure of CNN. The equation of one standard convolution layer is formulated by Eq. (11):

(11) $$X^{conv}=conv1D\left(W^{conv},X\right)\;$$

Where $X^{conv}\text{,}$ $W^{conv}$, correspondingly denotes the output vector, weighted matrix of convolutional layer, X indicates the sensors input, and conv1 D indicates the 1D convolutional operator. The hyperparameter of convolutional layer is the length of kernel $L_{k}$ representing the count of neighboring data points aggregated, and the amount of kernel $N_{k}$ representing the amount of local features extracted.

Then, $X^{conv}$ is fed into the LSTM layer that exploits data at many preceding time steps for perceiving insight into current time step, represented as “long‐term dependency”. Introducing L a classical linear conversion of integration of $X_{t}^{conv}$ with $N_{k}$ feature at t time step and resultant of hidden state $h_{t-1}$ with $N_{h}$ features at prior step:

(12) $$L\left(h_{t-1},X_{t}^{conv}\right)=W\left[h_{t-1},X_{t}^{conv}\right]+b\text{,}$$

In Eq. (12), W and b denote the weighted matrix and bias vector; it can be noteworthy that the amount of features of L is equivalent to that of hidden output h. All the cells of LSTM include 3 gates such as forget gate $f_{f}$, input gate $f_{i}$, and output gate $f_{0}$, that include nonlinear sigmoid function σ to a linear conversion L as follows:

$$f_{f}=\sigma \left(L_{f}\left(h_{t-1},X_{t}^{conv}\right))h_{t-1},X_{t}^{conv}\right))L_{f}$$

(13) $$f_{i}=\sigma \left(L_{i}\left(h_{t-1},X_{t}^{conv}\right)\right)\;$$

$$f_{0}=\sigma \left(L_{o}\left(h_{t-1},X_{t}^{conv}\right)\right)$$

At the same time, a novel candidate of data produced at t time step can be evaluated by the tanh activation function to linear conversion of concatenation $\left[h_{t-1};{\;X}_{t}^{conv}\right]$:

(14) $$C_{t}=\tanh\left(L_{c}\left(h_{t-1},X_{t}^{conv}\right)\right)\text{,}$$

Next, the candidate enters LSTM cells:

(15) $$s_{t}=\left(f_{f}⊙h_{t-1}\right)⊕\left(f_{i}⊙C_{t}\right)\text{,}$$

and hidden output of LSTM cell at t time step can be evaluated at the output gate:

(16) $$h_{t}=f_{0}⊙s_{t}\text{.}$$

Where ⊕ and ⊙ correspondingly represents component-wise addition and multiplication of two vectors. As soon as input data enter a network, it can be split into fixed‐length segments, and then the IDCNN layer extracts local connections amongst their surrounding points and data points beforehand, feeding to the memory cell of LSTM where long‐term dependency is recognized and preserved over time. During this hybrid DL structure, the hyperparameter that needs to be further defined is the size of hidden output $N_{h}$, amount of kernels $N_{k}$, and the kernel length $L_{k}$ in the convolutional layer at all the LSTM cells.

Finally, the RSA adjusts the hyperparameter values of the HDL model. The highly coordinated and cooperative hunting method demonstrated by the crocodiles includes encircling the target, and hunting has been an inspiration for the current reptile search algorithm RSA [21].

(17) $$X=\left[\begin{array}{ccccc} x_{1,1} & \ldots & x_{1,j} & x_{1,n-1} & x_{1,n}\\ x_{2,1}\; & \ldots & x_{2,j} & \ldots & x_{2,n}\\ \ldots & \ldots & x_{i,j} & \ldots & \ldots \\ \vdots & \vdots & \vdots & \vdots & \vdots \\ x_{N-1,1} & \ldots & x_{N-1,j} & \ldots & x_{N-1,n}\\ x_{N,1} & \ldots & x_{N,j} & x_{N,n-1} & x_{N,n} \end{array}\right]\;$$

The initialization stage begins with generating X matrix of random solution $x_{i,j}$ based on Eq. (17), where n denotes the dimensionality of specific problem, i represents the index of the individual, j shows its existing location, andN represents the overall amount of individuals.

(18) $$\chi_{ij}=rand\times \left(UB-LB\right)+LB,j=1,2,\ldots \text{,}n$$

Eq. (18) produces random individuals. Now, rand represents the arbitrary integer within the range, and LB and UB represent the lower and upper bounds of searching spaces. The search process was split into two major procedures (neighboring prey, afterwards the attack) accompanied by the 4 distinct behaviors for emphasizing exploration and exploitation. Exploration exploits 2 walking strategies demonstrated by crocodiles: stomach walk and elevated walk. The key objective of the crocodile is to extend the searching region and helps for the next hunting stage. The elevated walk method can be used if $t\leq \frac{T}{4}$, whereas the stomach walk is triggered if $t>\frac{T}{4}$ and $t\leq 2\frac{T}{4}\text{.}$ Eq. (19) is accountable for updating the position of crocodile:

(19) $$x\left(i,j\right)\left(t+1\right)=\left\{ \begin{array}{l} Best_{j}\left(t\right)\times -\eta_{\left(i,j\right)}\left(t\right)\times \beta -R_{\left(i,j\right)}\left(t\right)\times rand,t\leq \frac{T}{4}\\ Best_{j}\left(t\right)\times \chi_{\left(r_{1},j\right)}\times ES\left(t\right)\times rand,t>\frac{T}{4}\;and\;t\leq 2\frac{T}{4} \end{array}\right.$$

(20) $$\eta_{\left(i,j\right)}=Best_{j}\left(t\right)\times P_{\left(i,j\right)}\;$$

In Eq. (19), T shows the maximal amount of iterations, $Best_{j}$ represents the present optimum individual at j-th position, and t denotes the ongoing iteration. The hunting operator $\eta_{\left(i,j\right)}$ was determined by Eq. (20), where β shows the sensitive parameter fixed at 0.1, which governs the exploration performance.

The searching space was shrunk by using the reduction function, determined using Eq. (21), where $r_{1}$ denotes a random integer ranging from 1 to N, $x_{r1,j}$ signifies the $i_{th^{l}}s$ solution random location, and e represents a smaller value.

(21) $$R_{\left(i,j\right)}=\frac{Best_{j}\left(t\right)-\chi_{\left(r_{1^{\prime }}j\right)}}{Best_{j}\left(t\right)+\epsilon }\;$$

Eq. (22) evaluates the probability ratio, named "Evolutionary Sense", that arbitrarily alternates in [−2, 2] as round passes by:

(22) $$ES\left(t\right)=2\times r_{2}\times \left(1-\frac{1}{T}\right)\;$$

Where $r_{2}$ indicates the arbitrary value inside.

Eq. (23) define the percentage difference between the position of the observed and best‐obtained individual:

(23) $$P_{\left(i,j\right)}=\alpha +\frac{\chi_{\left(i,j\right)}-M\left(\chi_{i}\right)}{Best_{j}\left(t\right)\times \left(UB_{\left(j\right)}-LB_{\left(j\right)}\right)+\epsilon }\;$$

In Eq. (23), α denotes the sensitive variable, with the predetermined value 0.1, which controls the fluctuations amongst possible individuals appropriate for co-operated hunting. The corresponding upper and lower boundaries of the $j_{th}$ position were indicated as $UB_{\left(j\right)}$ and $LB_{\left(j\right)}\text{.}$.

The average location M(X) of ith individual was expressed as follows.

(24) $$M\left(\chi_{i}\right)=\frac{1}{n}\sum_{j=1}^{n}\chi_{\left(i,j\right)}\;$$

The RSA exploitation process is divided into hunting coordination (if $t\leq 3\frac{T}{4}$ and $t>\frac{T}{2})$ and cooperation (ift≤T and $t>3\frac{T}{4})$ technique, aims to strengthen the local investigation of the search realm and closer to the optimum individual. The hunting behavior shown by the crocodile has been expressed as.

(25) $$x\left(i,j\right)\left(t+1\right)=\left\{ \begin{array}{l} Best_{j}\left(t\right)\times P_{\left(i,j\right)}\left(t\right)\times rand,t\leq 3\frac{T}{4}\;and\;t>\frac{T}{2}\\ Best_{j}\left(t\right)-\eta_{\left(i,j\right)}\left(t\right)\times e-R_{\left(i,j\right)}\left(t\right)\times rand,t\leq T\;and\;t>3\frac{T}{4} \end{array}\right.$$

The basic RSA shows the time complexity of the O(N×(T×D+1 where N indicates the candidate counts, T represents the round counts, and D denotes the dimensional of solution spaces. The RSA method creates a fitness function (FF) to make superior classifier result. It explains a positive integer to exemplify the good performance of candidate outcomes. During this effort, the minimizing of classifier error rate was supposed that FF is formulated in Eq. (26).

(26) $$fitness\left(x_{i}\right)=ClassifierErrorRate\left(x_{i}\right)=\frac{number\;of\;misclassified\;samples}{Total\;number\;of\;samples}*100$$

## 4 Experimental Evaluation

**Fig. 3:**
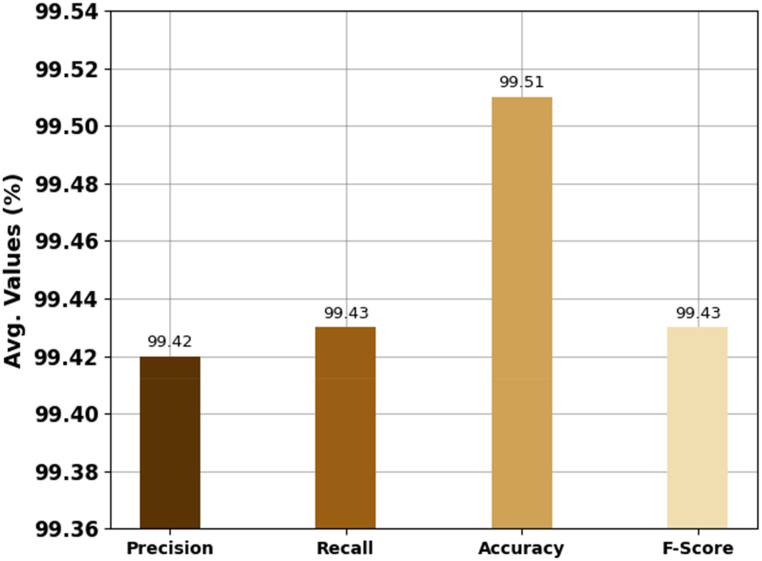
Average outcome of SLDC-RSAHDL approach.

In this section, the SLR performance of the SLDC-RSAHDL technique is studied using the ASL alphabet dataset from Kaggle repository [22]. The database has a group of images of alphabets in American Sign Language, divided into 29 folders that expose several classes. Table 1 and Fig. 3 offer a detailed recognition result of the SLDC-RSAHDL technique under 29 classes. The results indicate that the SLDC-RSAHDL technique performs proficiently in each class. At the same time, it is noticed that the SLDC-RSAHDL technique accomplishes effectual outcomes with average $prec_{n}$ of 99.42 %, $reca_{l}$ of 99.43 %, $accu_{y}$ of 99.51 %, and $F_{score}$ of 99.43 %.

**Table 1:** Classifier outcome of SLDC-RSAHDL approach under 29 classes.

Sign	Precision	Recall	Accuracy	F-Score	Sign	Precision	Recall	Accuracy	F-Score
A	99.25	99.80	99.65	99.69	P	99.57	99.33	99.58	99.32
B	99.39	99.45	99.70	99.20	Q	99.53	99.41	99.43	99.25
C	99.52	99.36	99.49	99.75	R	99.43	99.20	99.55	99.21
D	99.25	99.68	99.43	99.49	S	99.53	99.57	99.62	99.54
E	99.45	99.22	99.40	99.49	T	99.50	99.22	99.21	99.75
F	99.41	99.56	99.75	99.41	U	99.27	99.34	99.72	99.55
G	99.53	99.21	99.31	99.35	V	99.26	99.66	99.54	99.38
H	99.33	99.51	99.37	99.32	W	99.30	99.34	99.45	99.49
I	99.77	99.34	99.73	99.52	X	99.43	99.45	99.46	99.70
J	99.48	99.24	99.39	99.26	Y	99.27	99.53	99.74	99.30
K	99.27	99.49	99.36	99.32	Z	99.44	99.36	99.70	99.34
L	99.45	99.22	99.49	99.57	Space	99.51	99.72	99.64	99.28
M	99.76	99.43	99.33	99.66	Nothing	99.26	99.58	99.58	99.63
N	99.20	99.48	99.73	99.28	Delete	99.30	99.28	99.20	99.31
O	99.65	99.42	99.21	99.20	**Average**	**99.42**	**99.43**	**99.51**	**99.43**

**Fig. 4:**
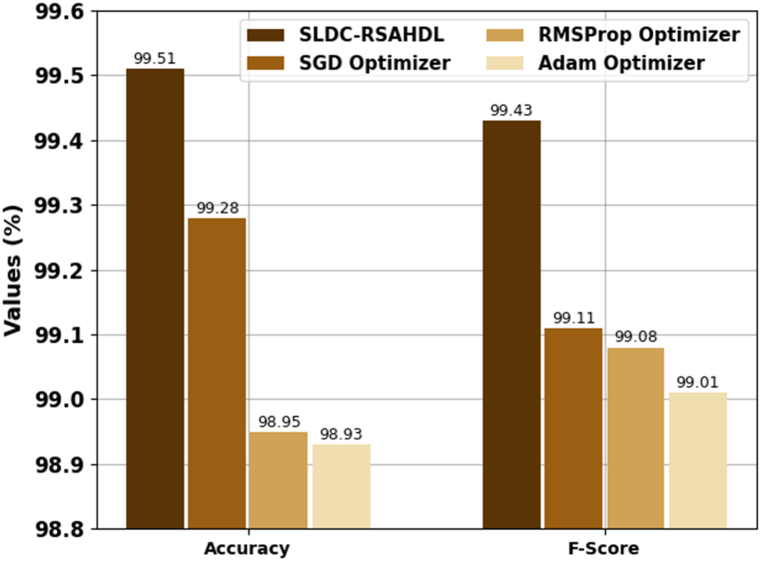
$Accu_{y}$ and $F_{score}$ outcome of SLDC-RSAHDL approach.

**Fig. 5:**
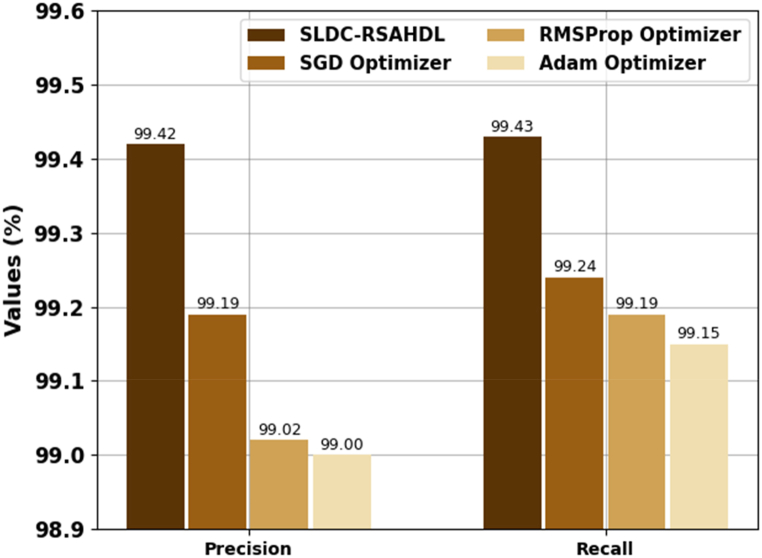
$Prec_{n}$ and $Reca_{l}$ outcome of SLDC-RSAHDL approach.

Table 2 and Fig. 4, Fig. 5 reports a brief recognition outcome of the SLDC-RSAHDL approach with other optimizers. The experimental values highlighted that the RMSProp optimizer and Adam optimizers had reached almost nearer performance with $accu_{y}$ of 98.95 % and 98.93 %, respectively. Along with that, the SGD optimizer gains considerable outcomes with $accu_{y}$ of 99.28 %, $prec_{n}$ of 99.19 %, $reca_{l}$ of 99.24 %, and $F_{score}$ of 99.11 %. However, the SLDC-RSAHDL technique resulted in enhanced performance with $accu_{y}$ of 99.51 %, $prec_{n}$ of 99.42 %, $reca_{l}$ of 99.43 %, and $F_{score}$ of 99.43 %.

**Table 2:** Recognition outcome of SLDC-RSAHDL approach with distinct measures.

Methods	$Accu_{y}$	$Prec_{n}$	$Reca_{l}$	$F_{Score}$
SLDC-RSAHDL	99.51	99.42	99.43	99.43
SGD Optimizer	99.28	99.19	99.24	99.11
RMSProp Optimizer	98.95	99.02	99.19	99.08
Adam Optimizer	98.93	99.00	99.15	99.01

**Fig. 6:**
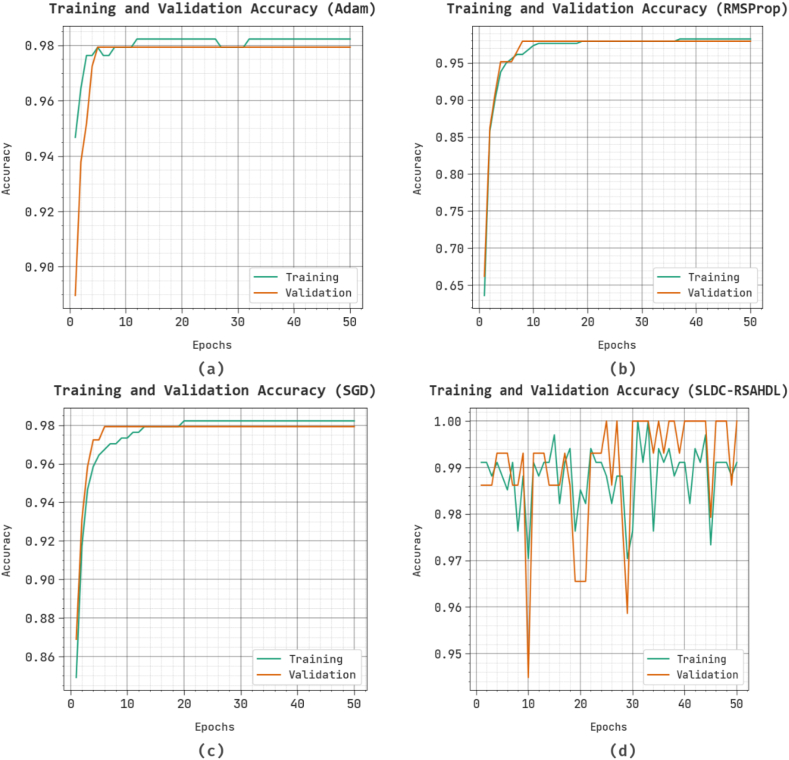
Accuracy curve of other existing approaches.

Fig. 6 inspects the accuracy of other existing techniques during the training and validation process on test dataset. The figure stated that the other existing techniques reach enhancing accuracy values over increasing epochs. Moreover, the increasing validation accuracy over training accuracy exposed those other existing methods that learn effectively on the test dataset.

**Fig. 7:**
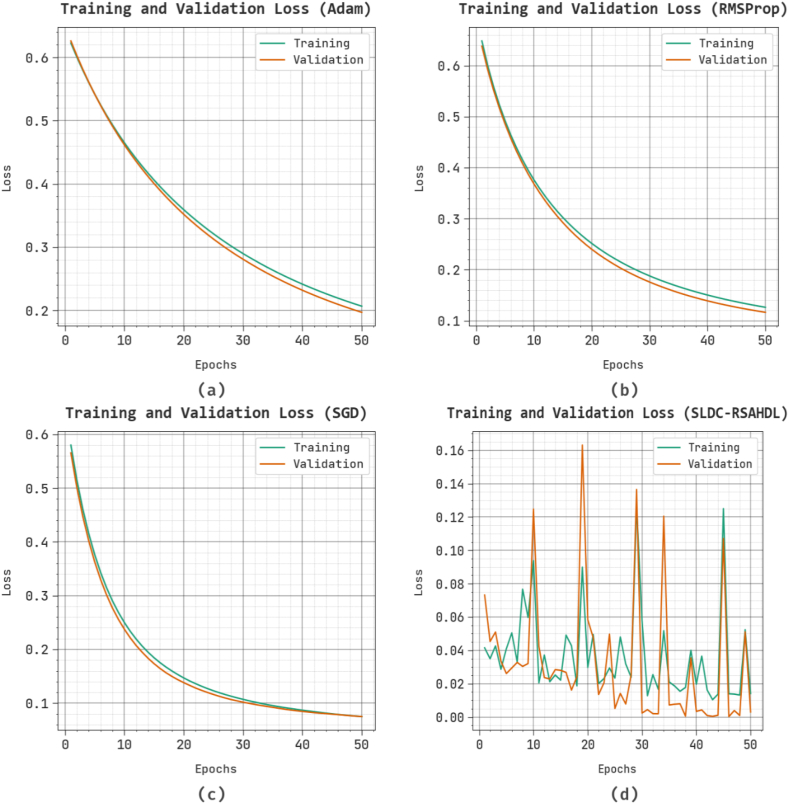
Loss curve of other existing approaches.

The loss investigation of other existing systems at the time of training and validation is exhibited on the test dataset in Fig. 7. The outcomes inferred that other existing methods gain closer values of training and validation loss. It is clear that other existing techniques learn effectively on the test dataset.

**Fig. 8:**
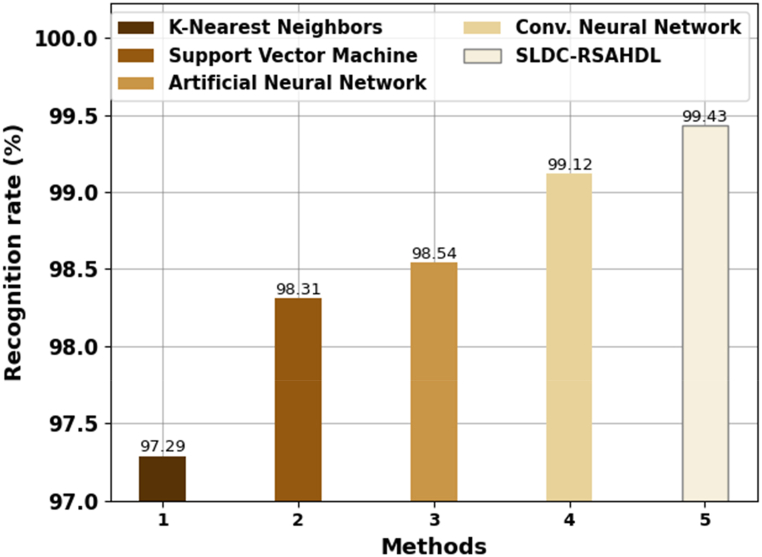
RR analysis of SLDC-RSAHDL approach with other algorithms.

Table 3 reports an overall comparison analysis of the SLDC-RSAHDL technique in terms of recognition rate (RR) and computation time (CT) [23]. In Fig. 8, a comparative RR investigation of the SLDC-RSAHDL technique with other models was performed. The results imply that the KNN model resulted from ineffective outcomes with minimal RR of 97.29 %. At the same time, the SVM and ANN models have accomplished considerably enhanced performance with closer RR of 98.31 % and 98.54 % respectively. Concurrently, the CNN model accomplishes reasonable RR of 99.12 %. But the SLDC-RSAHDL technique reaches higher performance with RR of 99.43 %.

**Table 3:** Comparative outcome of SLDC-RSAHDL system with other techniques.

Methods	Recognition rate (%)	Computation Time (min)
K-Nearest Neighbors	97.29	16.84
Support Vector Machine	98.31	15.10
Artificial Neural Network	98.54	14.36
Conv. Neural Network	99.12	11.26
SLDC-RSAHDL	99.43	6.14

**Fig. 9:**
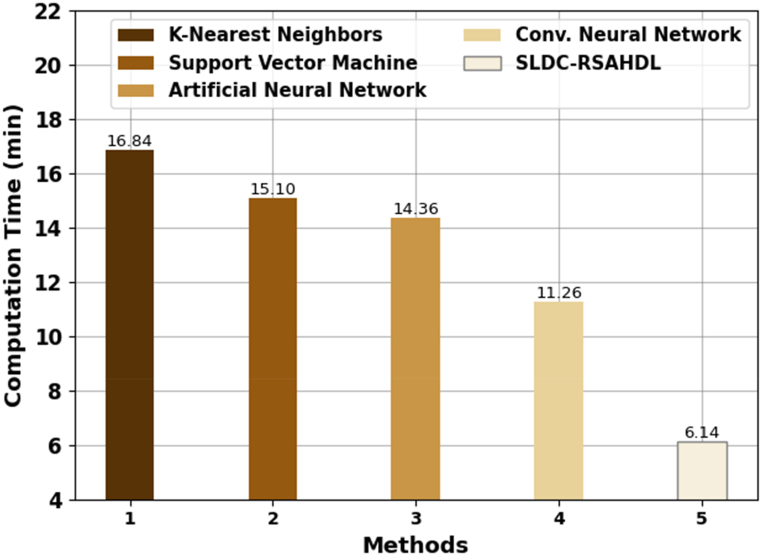
CT analysis of SLDC-RSAHDL approach with other algorithms.

In Fig. 9, a comparative CT examination of the SLDC-RSAHDL approach with other techniques was performed. The outcomes inferred that the KNN system resulted from ineffective outcomes with maximal CT of 16.84min. Besides, the SVM and ANN algorithms have obtained considerably superior performance with closer CTs of 15.10min and 14.36min. Finally, the CNN method reaches reasonable CT of 11.26min. However, the SLDC-RSAHDL system attains effectual performance with CT of 6.14min.

From the detailed results and discussion, it can be concluded that the SLDC-RSAHDL algorithm reaches effectual performance on the SLR process.

## 5 Conclusion

In this study, we have introduced a novel SLDC-RSAHDL technique for automated detection and classification of sign language using the DL and metaheuristic optimization algorithms. It follows a four-stage process: MobileNet feature extraction, MRFO based hyperparameter tuning, HDL based SLR, and RSA based parameter tuning. The design of the MRFO and RSA algorithms assists in the effectual selection of the hyperparameters related to the MobileNet and HDL models, which results in improved detection rate. The experimental result analysis of the SLDC-RSAHDL technique on sign language dataset demonstrates the improved performance of the SLDC-RSAHDL technique over other recent DL algorithms. In the future, the detection performance of the SLDC-RSAHDL technique was boosted by the fusion-based ensemble models' design.

## Data Availability Statement

The data used in this article was not collected from any public repository. The data collected as responses for this study was collected from individuals working in the case organization.

## Ethics approval

This article does not contain any studies with human participants performed by any of the authors.

## Consent to Participate

Not applicable.

## Funding details

None.

## Informed Consent

Not applicable.

## CRediT authorship contribution statement

**Hadeel Alsolai:** Conceptualization, Data curation, Funding acquisition, Methodology, Writing - original draft. **Leen Alsolai:** Conceptualization, Writing - original draft, Writing - review & editing. **Fahd N. Al-Wesabi:** Conceptualization, Writing - original draft, Writing - review & editing. **Mahmoud Othman:** Conceptualization, Methodology, Writing - original draft, Writing - review & editing. **Mohammed Rizwanullah:** Methodology, Software, Writing - original draft, Writing - review & editing. **Amgad Atta Abdelmageed:** Conceptualization, Data curation, Validation, Writing - original draft, Writing - review & editing.

## Declaration of Competing interest

The authors declare that they have no conflict of interest. The manuscript was written through contributions of all authors. All authors have given approval to the final version of the manuscript.
